# Polybutadiene Vitrimers with Tunable Epoxy Ratios: Preparation and Properties

**DOI:** 10.3390/polym13234157

**Published:** 2021-11-28

**Authors:** Liqian Zhu, Li Xu, Suyun Jie, Bogeng Li

**Affiliations:** State Key Laboratory of Chemical Engineering, College of Chemical and Biological Engineering, Zhejiang University, Hangzhou 310027, China; liqianzhu@zju.edu.cn (L.Z.); 11628036@zju.edu.cn (L.X.); jiesy@zju.edu.cn (S.J.)

**Keywords:** polybutadiene vitrimer, epoxidized PB, disulfide exchange, crosslinking density, network topology

## Abstract

Traditional crosslinked diene rubber has excellent thermal–mechanical properties and solvent resistance, yet it is incapable of being recycled via universal molding or injecting. Vitrimers, a new class of covalently crosslinked polymer networks, can be topologically rearranged with the associative exchange mechanism, endowing them with thermoplasticity. Introducing the concept of vitrimers into crosslinked networks for the recycling of rubbers is currently an attractive research topic. However, designing tailored rubber vitrimers still remains a challenge. Herein, polybutadiene (PB) vitrimers with different structures were prepared via partial epoxidation of double bonds and ring-opening esterification reactions. Their mechanical and relaxation properties were investigated. It was found that the increasing crosslinking density can increase tensile strength and activation energy for altering the network topology. The influence of side-group effects on their relaxation properties shows that an increase in the number of epoxy groups on the polybutadiene chain can increase the chance of an effective exchange of disulfide units. This work provides a simple network design which can tune vitrimer properties via altering the crosslinking density and side-group effects.

## 1. Introduction

Traditional vulcanized rubbers exhibit excellent thermal stability, creep resistance, and solvent resistance due to stable three-dimensional covalently crosslinked networks. However, the irreversibility of chemical bonds prevents their recycling. Thermoplastic elastomers can be recycled, because their linear structure permits melting at high temperatures. However, their weak intermolecular forces lead to poor heat and solvent resistance, which also limits their applications [1,2].

Vitrimers, pioneered by Leibler and his group [3], are a new class of crosslinked polymers with great malleability, promising in the combination of recycling and high performance. The introduction of dynamic exchange bonds allows the polymer network to be rearranged while maintaining the network integrity, which gives them the ability to undergo repairing and recycling [4]. At room temperature, vitrimers possess a relatively strong crosslinked structure because of the “frozen” polymer networks, whereas, at high temperatures, they can flow via network rearrangement. Furthermore, their viscosity is controlled by exchange reactions; therefore, it exhibits a gradual decrease, following the Arrhenius law [5]. The vitrimeric rheological behavior endows vitrimers with solid-state plasticity, avoiding complex molds or precise temperature windows [6,7]. Due to these charming characteristics, their potential applications in the fields of modification of thermoplastic polymers and thermo-plasticization of thermoset polymers have attracted growing attention.

The introduction of vitrimers endows thermoplastic elastomers with stronger intermolecular forces without compromising their processing properties. Caffy et al. [8] transformed polyethylene into vitrimers through reactive extrusion to improve their creep resistance. Yang et al. [9] reported polyolefin elastomer (POE) vitrimers. The dynamic covalently crosslinked network based on boronic ester bonds significantly improved the mechanical properties, as well as thermal and solvent resistance, without impairing their recycling ability. Kar et al. [10] reported a simple strategy to produce vitrimers from polyolefins, and the thermo-triggered bond exchange endowed vitrimers with shape memory characteristics.

Nevertheless, it is more challenging to achieve the thermo-plasticization of thermoset polymers. When using dynamic chemistry to realize the recycling of thermosets, the balance between the stability and reversibility of crosslinks needs to be considered more carefully. Rubbers, a special thermoset polymer with unique entropy elasticity, are widely used in tire, sealing, and damping materials [1]. So far, various dynamic covalent linkages have been developed for the green recycling of rubbers, including transesterification [11,12,13,14], transamination [15], disulfide exchange [16], boronic ester exchange [17,18,19,20], olefin metathesis [21,22,23], and imine chemistry [24,25]. However, there have been few reports about the effects of crosslinking density on the overall performance of vitrimers. Hayashi et al. [26,27] explored the influence of crosslinking density on bond exchange properties for polyester vitrimer elastomers. Raymond et al. [28] synthesized a series of polyimine elastomeric vitrimers to study the influence of crosslinking density on their relaxation properties. The influence of network structure on the properties of rubber vitrimers has not been the focus of investigations and still remains to be explored.

Unsaturated rubber is some of the most versatile commercial rubber available, and enabling it to be recycled through vitrimer chemistry has gradually become a research hotspot in sustainable development for the rubber industry. Olefin metathesis is a simple and effective tool to make covalently crosslinked rubber malleable and self-healing. However, the designed vitrimer is prone to creep, and the deactivation of the catalyst during processing may impair the malleability of networks [5]. In fact, it is not easy to design a vitrimer reliant on reversible carbon–carbon bonds, except for olefin metathesis. Therefore, the chemical modification of carbon–carbon double bonds is significant for unsaturated rubber vitrimers [29]. Epoxidation is one such powerful method, and the epoxy group is a common reactive site to form vitrimers [3]. Given its universality, transesterification is a prototypical choice for epoxy-functionalized rubber vitrimers. Various recyclable rubber vitrimers enabled by transesterification have been reported [30,31,32]. Most require catalysts to activate the exchange, and their epoxy ratios are fixed. Liu et al. [33] synthesized a catalyst-free polybutadiene vitrimer based on imine bonds via further modification of epoxidized polybutadiene to explore the influence of the precursor molecular weight. However, the strategy of end-group crosslinking is unlike conventional crosslinked rubber.

Disulfide exchange is quite favored in the design of commercial rubber vitrimers for its charming features involving multi-stimulus responsiveness and mild conditions. Imbernon et al. [34] introduced disulfide bonds into epoxidized natural rubber using dithiodibutyric acid as the crosslinker to synthesize covalently crosslinked networks with reprocessable capability. Xiang et al. [35,36] introduced Cu^2+^ catalysts into sulfur vulcanized rubbers, and the activated disulfide exchange endowed them with self-healing and recycling abilities. In addition, they reported a simple strategy to prepare photo-crosslinkable polybutadiene rubbers, and disulfide exchange triggered via UV irradiation made the chemically crosslinked networks self-healable and reprocessable [16]. Cheng et al. [37] introduced dithiodibenzoic acid and dithiodianiline into sulfur crosslinked epoxidized natural rubber to promote disulfide exchange, and the obtained elastomer exhibited great self-healing and recycling capabilities. Odriozola et al. [38,39] demonstrated the preparation of dynamically crosslinked poly(urea–urethane) elastomers. The aromatic disulfide linkages gave the elastomer self-healing and reprocessing abilities. Unfortunately, these reports did not carefully consider the effect of topological structure.

Herein, we synthesized epoxidized polybutadiene vitrimers with tunable epoxy ratios to explore the relationship between the network and the final properties of vitrimers. First, polybutadiene with different epoxidation degrees was prepared via reaction-controlled phase-transfer catalysis. According to our previous work [40], the quantitative epoxidation of polybutadiene rubber with a high *cis*-1,4 structure could be achieved using H_2_O_2_ and a phase-transfer catalyst. Moreover, the reaction was selective, only occurring in the main chains. Thus, a series of PB vitrimers were prepared from the ring-opening esterification reactions between epoxy groups and disulfide-containing dicarboxylic acids to study the relationship between networks and their mechanical and relaxation properties.

## 2. Materials and Methods

### 2.1. Materials

Commercially available polybutadiene rubber (BR 9000; Mn = 120,000 g/mol; *cis*-1,4 = 96.7%; M_w_/M_n_ = 4.2) was provided by Zhejiang Transfar Co., Ltd. (Hangzhou, China). Methanol, 1,2-dichloroethane (DCE), tetrahydrofuran (THF), and aqueous hydrogen peroxide (H_2_O_2_, 30 wt.%) were purchased from Sinopharm Chemical Reagent Co., Ltd. (Shanghai, China). 4,4′-Dithiodibutyric acid (DTBA, 95%), 3,3′-dithiodipropionic acid (DTPA, 99%) and 1,2-dimethylimidazole (DMI, 97%) were purchased from Sigma-Aldrich (St. Louis, MO, USA). Decanedioic acid (DA, 99%) was obtained from Shanghai Macklin Biochemical Co., Ltd. (Shanghai, China). The phase-transfer catalyst was self-made according to a previous study of our group [40].

### 2.2. Preparation of Polybutadiene Vitrimers (PB Vitrimers) 

The epoxidized polybutadiene (EPB) with different epoxy ratios (10, 15, 25, and 35 mol.% relative to the diene content) was prepared as described in [40] and Section 1 of the Supplementary Materials. And it was named as EPBx with x the epoxy ratios. 

The required number of crosslinking agents (disulfide-containing dicarboxylic acid) and DMI were successively added into the EPB solution in THF (10 wt.%) with magnetic stirring at room temperature. After 2 h, the mixture was heated to 40 °C to remove the solvent, followed by vacuum-drying at 30 °C overnight. Finally, the dried sample was molded by compression at 150 °C for the appropriate time determined by the crosslinking kinetics. The quantity of crosslinking agents was controlled at 0.5, 1.0, and 1.5 mol.% (relative to the monomers of EPBx), and the amount of DMI acting as an accelerator was 25 mol.% (relative to the carboxyl groups). The compositions of PB vitrimers are summarized in Table 1.

For comparison, DA without the disulfide bonds was chosen as the crosslinking agent, and the preparation of the control sample was similar to that of the EPB vitrimers. The epoxy ratio of the chosen EPBx was 25%. The amount of DA was fixed at 1.0 mol.% (relative to the monomers of EPB25), and the content of DMI was 25 mol.% (relative to the carboxyl groups). The obtained sample was named EPB25-DA.

### 2.3. Characterization 

The microstructure of EPB was characterized by ^1^H-NMR spectroscopy (AVANCEIII 400 MHz, Bruker, Karlsruhe, Germany), using CDCl_3_ as the solvent. FT-IR spectroscopy was performed on a Nicolet 5700 spectrometer equipped with a Smart SpeculATR accessory (Thermo Fisher, Waltham, MA, USA) in the range of 4000–650 cm^−1^ for 32 scans at a resolution of 4 cm^−1^. Temperature-dependent FT-IR spectra were recorded on a Nicolet IS 50 spectrometer equipped with a temperature controller and an integrating sphere for 64 scans at a resolution of 8 cm^−1^. Raman spectroscopy was carried out on a Horiba Scientific LabRAM HR Evolution spectrometer (Horiba, Longjumeau, France) using a laser with a wavelength of 785 nm.

The crosslinking kinetics were followed by a HAAKE RS6000 rheometer (Haake Technik GmbH, Vreden, Germany). Following rapid heating from 30 °C to 150 °C, the temperature was kept at 150 °C for curing (plate–plate geometry, 20 mm diameter, 1 Hz frequency, CD mode, 0.1% strain). The crosslinking density was evaluated by equilibrium swelling experiments. The samples were immersed in toluene at room temperature for 72 h and then vacuum-dried until constant weight. The swelling ratio (*Q*) was calculated according to Equation (1).

(1) $$Q=\frac{\left(m_{1}-m_{2}\right)}{m_{2}}.$$

The volume fraction of rubber in the swollen sample (*v_r_*) was calculated according to Equation (2) [41].

(2) $$v_{r}=\frac{\frac{m_{2}}{\rho_{r}}}{\frac{m_{2}}{\rho_{r}}+\frac{\left(m_{1}-m_{2}\right)}{\rho_{s}}},$$

where *m*_1_ and *m*_2_ are the weights of the swollen and dried sample, and *ρ_r_* and *ρ_s_* are the densities of the rubber and toluene, respectively. The crosslink density (*v_e_*) was calculated according to Equation (3) [42].

(3) $$\upsilon_{e}=-\frac{\ln(1-v_{r})+v_{r}+\chi v_{r}{}^{2}}{v_{s}(v_{r}{}^{\frac{1}{3}}-\frac{v_{r}}{2})},$$

where *χ* is the Flory–Huggins polymer–solvent interaction term (0.23 for PB), and *v_s_* is the molar volume of the solvent (106.5 cm^3^/mol for toluene) [33].

Thermogravimetric analysis (TGA) was performed on a TA-Q500 thermogravimetric analyzer (TA Instrument, Newcastle, DE, USA) from 50 °C to 600 °C at a heating rate of 10 °C·min^−1^ under nitrogen. Isothermal TGA was performed at 150 °C for 2 h under an air flow. The sample amounts for both tests were 3–4 mg. Differential scanning calorimetry (DSC) was conducted using a DSC 204HP differential scanning calorimeter (NETZSCH Group, Selb, Germany) under a nitrogen flow. The samples (6–8 mg) were maintained isothermally at 50 °C for 5 min to eliminate thermal history, and then cooled to −150 °C with a cooling rate of 10 °C·min^−1^, followed by a heating scan from −150 °C to 80 °C at a heating rate of 10 °C·min^−1^.

Dynamic mechanical analysis (DMA) was carried out on a TA Q800 dynamic mechanical analyzer (TA Instrument, Newcastle, DE, USA) in tensile mode (30 mm × 5 mm × 1 mm). The samples were scanned from −110 °C to 110 °C at a heating rate of 3 °C·min^−1^ (1 Hz frequency, 0.1% strain). Stress relaxation was conducted on a TA Q800 dynamic mechanical analyzer. After soaking for 5 min at the testing temperature, 1% constant strain was applied, and the stress change was monitored over time. Creep recovery experiments were determined by a TA Q800 dynamic mechanical analyzer. After soaking for 5 min at the testing temperature, 0.1 MPa constant stress was applied for 60 min. Then, 0 MPa stress was applied for another 60 min. The strain change was monitored over time. For the cyclic creep recovery experiment, 0.2 MPa constant stress for 20 min and 0 MPa stress for 10 min were loaded in each cycle. Thermodilatometry experiments were conducted on a TA Q800 dynamic mechanical analyzer from 20 °C to 210 °C at a heating rate of 3 °C·min^−1^, and 10^−2^ N static force was applied during scanning.

Tensile testing was characterized by a Zwick/Roell Z020 universal material testing machine (Zwick Armaturen GmbH, Ennepetal, Germany) with a tensile rate of 10 mm·min^−1^ at room temperature. The specimens were Type 4 dumbbell test species in the GB/T 528-2009 standard (matching the ISO 37-2005 standard) with approximately 1 mm thickness. The strain was measured via the distance between two fixtures instead of an extensometer. At least five specimens were provided for each material, and cyclic tensile testing was performed at 25%, 40% and 50% strain for five cycles. The recycling property was determined by the tensile testing. The samples were ground into small particles and then remolded at 150 °C for 30 min using a vulcanizer under 5 MPa pressure.

The shape-changing test was carried out to evaluate the plasticity. The sample (60 mm × 5 mm × 1 mm) was deformed by applying an external force and then put into an oven at 150 °C for 30 min.

For NMR characterizations, the sample was dissolved in CDCl_3_ to form a transparent homogeneous solution. For rheological testing, the uncured sample was molded by compression at 80 °C for 15 min (20 mm diameter, 1.2 mm thick). For FT-IR, Raman, DSC, TGA, equilibrium swelling, and shape-changing analyses, the samples were obtained from compression molding as described in Section 2.2. For tensile and DMA tests, the samples were obtained by cutting on the compression molded samples with cutters.

## 3. Results and Discussion

### 3.1. Design and Characterization of PB Vitrimers

**Figure 1:**
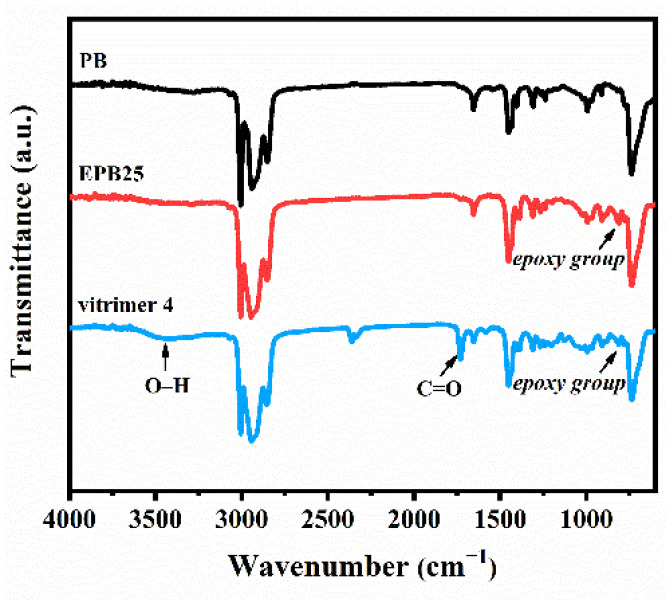
FT-IR spectra of PB, EPB25, and vitrimer 4.

Herein, the epoxidized polybutadiene was synthesized via reaction-controlled phase-transfer catalysis (Figure S1). Compared with original polybutadiene (PB), a new peak appeared at around 812 cm^−1^ in the EPB25, corresponding to the asymmetrical stretching vibration of the epoxy group (Figure 1). The new characteristic peak in the epoxidized polybutadiene at around 2.93 ppm was attributed to the protons of *cis*-1,4 epoxy groups (Figure S2). The experimental epoxidation degree measured by the peak integral was almost consistent with the theoretical value calculated by the addition amount of H_2_O_2_. Collectively, the results of FT-IR and ^1^H-NMR spectra confirmed the successful introduction of epoxy groups via reaction-controlled phase-transfer catalysis.

**Scheme 1:**
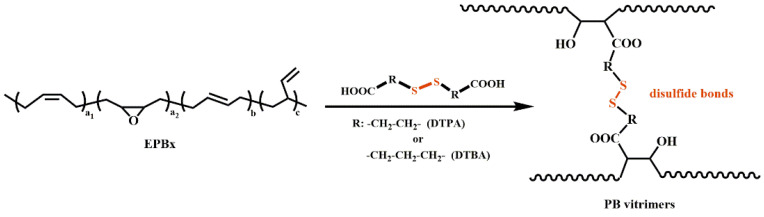
The formation of the PB vitrimers via the reaction between epoxy groups and carboxylic groups.

Exchangeable disulfide bonds could be introduced into the polymer network through the chemical reaction between epoxy groups in the polybutadiene backbone and carboxylic groups in the crosslinker (Scheme 1). Upon manipulating the network parameters including the epoxy ratio in polybutadiene chains, the length of crosslinkers, and the content of the crosslinker, a series of dynamically crosslinked networks with similar microstructures were constructed to study the relationship between the network structure and final properties of PB vitrimers.

FT-IR characterizations proved the existence of ester groups in PB vitrimers. Compared with epoxy-functionalized polybutadiene, the new peaks at 1730 cm^−1^ and 3400–3600 cm^−1^ in vitrimer 4 were ascribed to the stretching vibration of C=O and O–H, respectively (Figure 1). FT-IR spectra of the other vitrimers also exhibited similar results (Figure S3). Furthermore, the existence of disulfide bonds was confirmed by Raman spectra (Figure S4). The absorption peaks of S–S and C–S appeared only in the vitrimer 4, compared with the control sample, EPB25-DA [43]. These results demonstrated that the crosslinked polybutadiene networks with disulfide bonds were prepared successfully via esterification reactions.

The crosslinking of PB vitrimers was also proven by monitoring the change in storage modulus over time. As shown in Figure S5, all PB vitrimers exhibited similar crosslinking behavior. The initial decrease in storage modulus was due to rubber softening during the fast temperature rise [14]. At 150 °C, the dramatic increase in modulus indicated the occurrence of the crosslinking. Obviously, the increasing epoxidation degrees and crosslinker content accelerated the crosslinking reaction rate, because the increase in the concentration of reactive groups improved the chance of effective collision. The similar crosslinking reaction rate between vitrimer 4 and vitrimer 6 was due to their close concentration of reactive groups.

Crosslinking density, a key physical property of network structure, was measured through the equilibrium swelling experiments. All materials exhibited good stability in toluene due to the successful formation of covalently crosslinked networks, and their crosslinking density was assessed by means of the classical Flory–Rehner equation (Table 2). Obviously, increasing the content of DTBA or shortening the chain length of crosslinkers would result in an increase in the crosslinking density. It was also affected by the epoxidation degree. Increasing the epoxy ratio would slightly increase the crosslinking density.

### 3.2. Thermal Properties of PB Vitrimers

**Figure 2:**
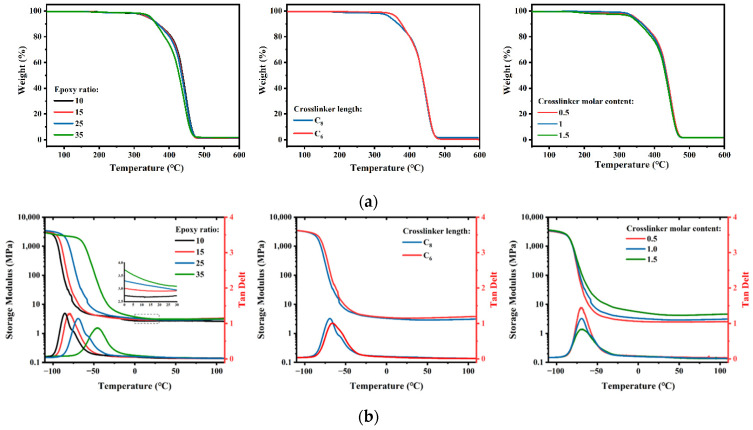

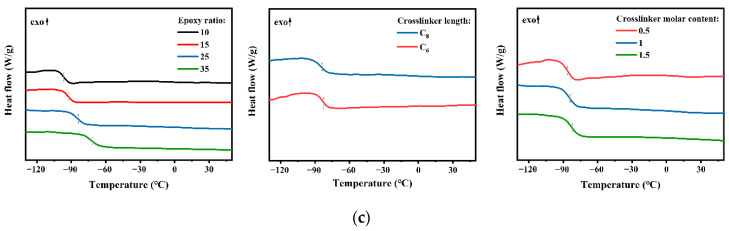
(**a**) TGA profiles, (**b**) DMA curves, and (**c**) DSC thermograms for PB vitrimers with the modification of network parameters.

The thermal stability of dynamically crosslinked networks was measured by TGA. The degeneration behavior was similar for all vitrimers, and the initial decomposition temperature (T_d_, the temperature of 5% mass loss) of each sample was above 330 °C, which demonstrated that the designed dynamically covalently crosslinked polybutadiene possessed excellent thermal stability (Figure 2a). Moreover, the isothermal TGA profiles determined at 150 °C for each vitrimer demonstrated their stability during processing (Figure S6).

The thermomechanical properties of PB vitrimers were demonstrated by DMA (Figure 2b). As expected, an increase in the crosslinking density generated an increase in the storage modulus of the rubbery plateau and the temperature of the maximum value in tan delta (T_δ_), because the increasing crosslinking density restricted the mobility of polymer chains. Interestingly, the increasing tendency was more obvious for PB vitrimers with different epoxidation degrees, although their change in crosslinking density was slight. The explanation might lie in the fact that, as the epoxidation degree increased, the polarity and hydrogen bonding increased. The glass transition temperatures (T_g_) obtained by DSC (Figure 2c) revealed a similar trend, which further proved the results measured by DMA. The detailed data for thermal properties of PB vitrimers are summarized in Table 2.

### 3.3. Mechanical Properties of PB Vitrimers

**Figure 3:**
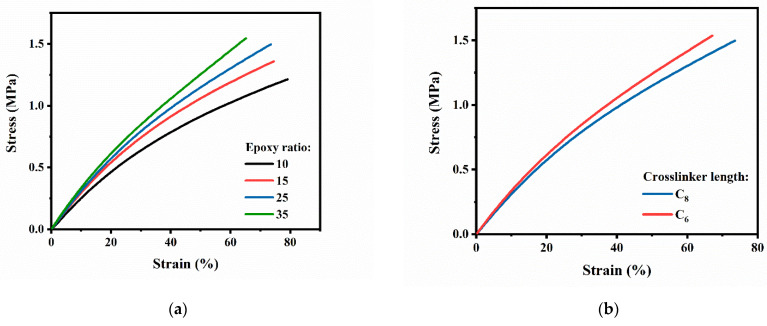

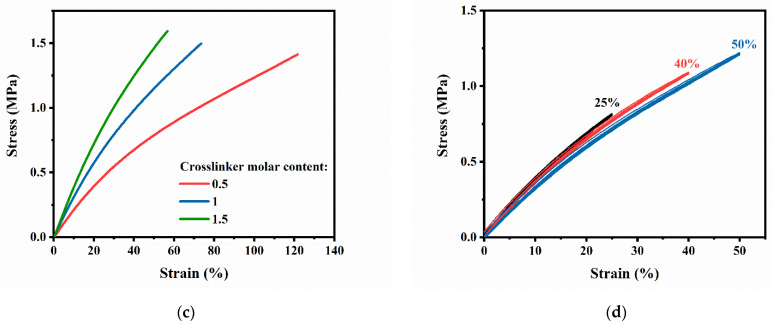
Typical stress–strain curves for PB vitrimers with the modification of network parameters: (**a**) epoxy ratio, (**b**) crosslinker length, and (**c**) crosslinker molar content; (**d**) stress–strain curves of vitrimer 4 for five repeated cycles.

Tensile experiments were conducted to evaluate the effect of crosslinked network architecture on mechanical properties for the designed vitrimers. Representative stress–strain curves of PB vitrimers are revealed in Figure 3a–c, and the detailed mechanical properties are summarized in Table S1. Obviously, the mechanical properties could be controlled by the change in network parameters. It could be further deduced that this control probably relied on the crosslinking density (Figure S7). The increasing crosslinking density improved tensile strength; however, it led to a decrease in elongation at break. This demonstrated the dependence of mechanical properties on network crosslinking density, similar to that of classical chemically crosslinked rubbers. On the other hand, as shown in Figure 3a, H-bonding interactions between epoxy and hydroxyl groups might improve the mechanical properties to some extent [39]. As shown in Figure 3d, the area of the hysteresis loops for vitrimer 4 was tiny, indicating that our system had an excellent elastic recovery ability owing to the covalently crosslinked network. In addition, the DTBA-cured polybutadiene vitrimer and DA-cured polybutadiene network possessed similar tensile properties (Figure S8), which confirmed the stability of the dynamically covalently crosslinked networks at room temperature. Collectively, the above results demonstrated the permanently crosslinked structure of PB vitrimers at room temperature, from macroscopical aspects.

### 3.4. Topological Network Rearrangements of PB Vitrimers

**Figure 4:**
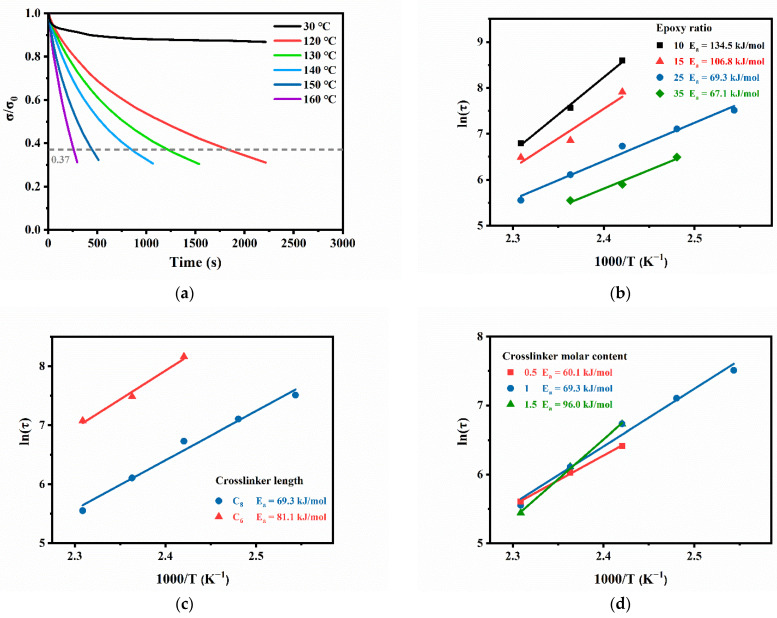
(**a**) Stress relaxation curves of vitrimer 4; linear fitting of the relaxation times by Arrhenius equation for EPB vitrimers with the modification of network parameters: (**b**) epoxy ratio, (**c**) crosslinker length, and (**d**) crosslinker molar content.

Owing to the activated disulfide exchange, PB vitrimers could alter network topology at high temperatures. Stress relaxation experiments were conducted to study the topological network rearrangement (Figure 4a and Figure S9). A slight relaxation at room temperature was observed for vitrimer 4, further confirming the stability of the dynamically covalently crosslinked network structure at room temperature. As the temperature increased, vitrimer 4 exhibited substantial relaxation behavior because of the thermoactivated disulfide exchange. In fact, the β-hydroxyl esters formed through the reaction between epoxy groups and carboxylic groups could also lead to stress relaxation via transesterification [3]. However, transesterification was excluded in our system due to the scarcity of free hydroxyl groups and transesterification catalysts [44,45]. The characteristic relaxation time (τ*) was defined as the time of 37% (1/e) of the initial stress. Obviously, the increasing temperature accelerated the relaxation rate, which followed the Maxwell model, i.e., the Arrhenius law (Equation (4)).

(4) $$\ln(\tau )=\frac{E_{a}}{RT}-\ln A.$$

The required activation energy (*E_a_*) was obtained through the linear fitting of the relaxation times and the reciprocal of corresponding temperature. As shown in Figure 4b–d, the relaxation process could be controlled by tunning the network structure. Shortening the chain length of crosslinkers or increasing the content of DTBA would increase the activation energy, whereas improving the epoxidation degree would decrease it.

**Figure 5:**
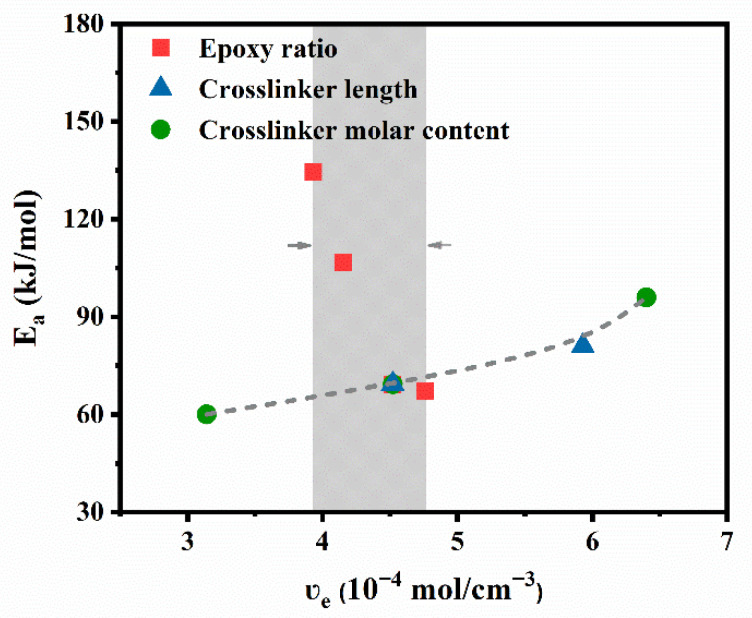
The relationship between activation energy and the crosslinking density.

The relationship between activation energy and the crosslinking density is depicted in Figure 5. As expected, increasing the crosslinking density led to an increase in the values of E_a_. This was mainly because, as the crosslinking density increased, the mobility of polybutadiene chains was limited [20], and more exchanges were required to achieve the network rearrangements [21]. Surprisingly, the opposite result was observed for the vitrimers with different epoxy ratios. Their values of activation energies decreased with an increase in crosslinking density. In fact, the distinction in their crosslinking density was small due to the same added content of DTBA. Therefore, it could be speculated that, in a narrow range of crosslinking density, the relaxation probably depended more on the side-group effects (i.e., epoxy groups) than on the crosslinking density. Hydrogen bonding around dynamic covalent bonds was in favor of the contact among disulfide bonds [46].

**Figure 6:**
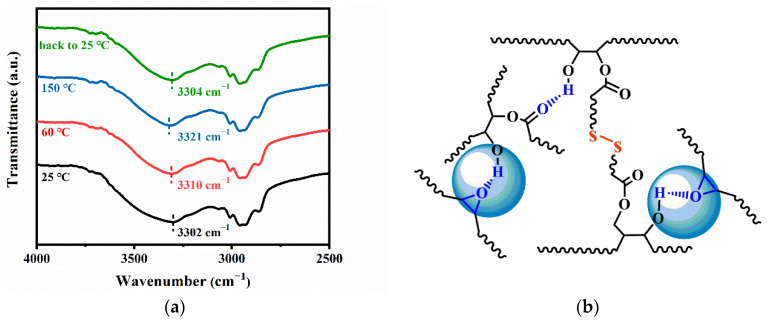
(**a**) FT-IR spectra of vitrimer 4 at different temperatures; (**b**) the illustration of hydrogen bonds in the network of PB vitrimers.

Temperature-dependent FT-IR spectra of vitrimer 4 are depicted in Figure 6a. The dissociation of hydrogen bonding made the peak ascribed to hydroxyl groups blue-shift to a higher wavenumber as the temperature increased [37]. When the temperature went back to room temperature, the peak shifted back to the virgin position owing to the reassociation of free hydroxyl groups. The change in the characteristic peak shift demonstrated the existence of hydrogen bonding in our system. Moreover, the increasing epoxidation degree strengthened the hydrogen bonding in vitrimer networks, as shown in Figure 6b, which promoted the proximity among the dynamic bonds [39]. On the other hand, the growth of epoxy groups as reactive sites led to a decrease in the average distance between crosslinks, which could be proven through the variation of the crosslinking density. In conclusion, it was reasonable to deduce that the increasing epoxy groups improved the chance of disulfide units approaching and colliding to achieve effective exchange, which consequently led to a reduction in E_a_.

**Figure 7:**
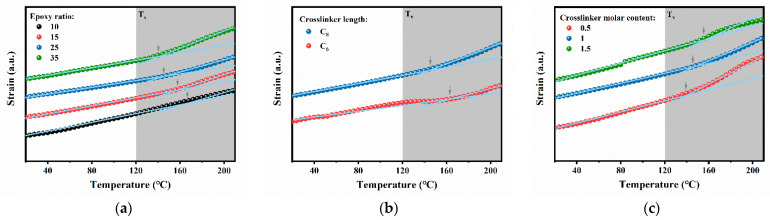
Thermodilatometry curves for PB vitrimers with the modification of network parameters: (**a**) epoxy ratio, (**b**) crosslinker length, and (**c**) crosslinker molar content.

The topological freezing transition temperature (Tv), a characteristic transition temperature of vitrimers in addition to the glass transition temperature, was determined by dilatometry experiments [47]. As shown in Figure 7, all vitrimers exhibited similar thermodilatometry behavior. In the first stage (from 20 °C to about 120 °C), the strain increased linearly, indicating the covalently crosslinked structure of networks. However, the slops of curves (i.e., the coefficient of thermal expansion) exhibited an abrupt rise due to the rearrangement of network topology via thermoactivated disulfide exchange, when the temperature was above 120 °C.

**Figure 8:**
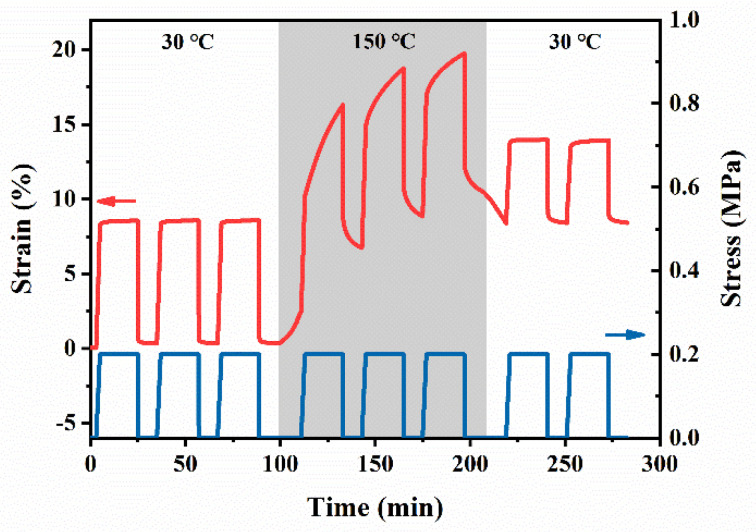
Cyclic strain recovery curves of vitrimer 4.

To further verify the thermo-responsive property of our prepared dynamically crosslinked networks, cyclic strain recovery experiments were conducted (Figure 8). At 30 °C, vitrimer 4 underwent elastic deformation with an external force and returned to the original state upon removing the applied force, confirming the good resilience of the designed vitrimer at room temperature, similar to permanently crosslinked rubber. When the temperature rose to 150 °C, the network showed a completely different viscoelastic response due to activated disulfide exchange. In addition to elastic deformation and thermal expansion, vitrimer 4 underwent viscous deformation upon the application of an external stress, unable to recover its original length. After the temperature returned to 30 °C, the frozen topological network resulted in the sample again behaving like traditional vulcanized rubber. The above results demonstrate that temperature could be used as a switch to manipulate the rearrangement of vitrimer networks.

**Figure 9:**
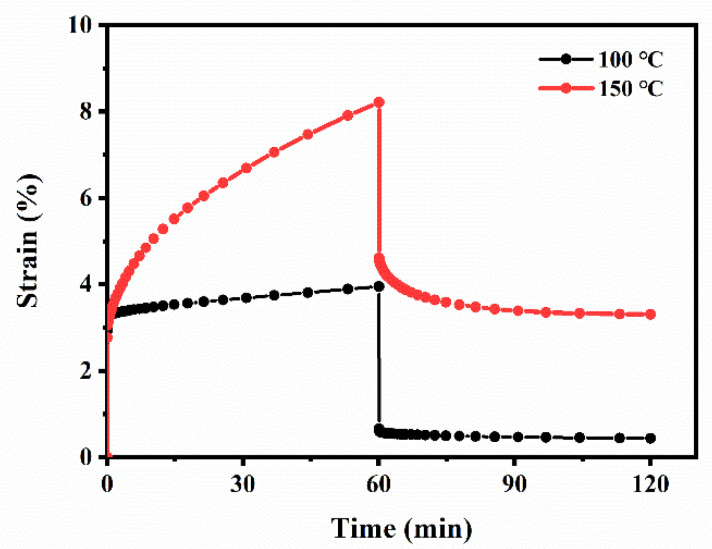
Creep recovery curves of vitrimer 4 at 100 °C and 150 °C.

Compared with thermoset polymers, vitrimers are prone to creep at high temperatures due to the network rearrangement caused by exchange reactions. To assess the creep resistance of PB vitrimers, creep recovery experiments were conducted (Figure 9 and Figure S10). The residual deformation of each vitrimer at 100 °C was below 1%, indicating their good creep resistance even at the high temperature of 100 °C. However, when the temperature went up to 150 °C, the accelerated network rearrangement led to significant deformations.

**Figure 10:**
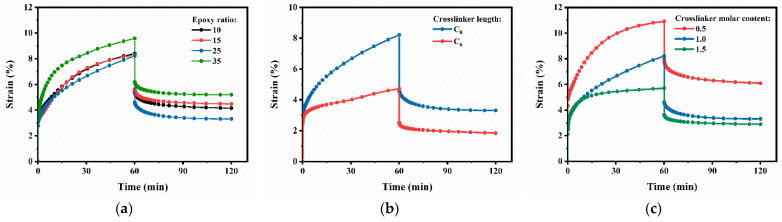
Creep recovery curves for PB vitrimers at 150 °C with the modification of network parameters: (**a**) epoxy ratio, (**b**) crosslinker length, and (**c**) crosslinker molar content.

The influence of network design upon creep resistance was studied (Figure 10). As expected, a decrease in the crosslinking density led to an increase in deformations. Furthermore, the increase in epoxidation degree also resulted in higher deformations, as seen in vitrimer 7, as epoxy groups could promote dynamic exchange. In general, the relaxation and creep properties of vitrimers represent a pair of contradictions. A reduction in relaxation time is beneficial for processing and recycling. On the other hand, a rapid rearrangement of network topology would restrict the creep resistance, which is against their long-term use. The permanent deformations and relaxation times of PB vitrimers at 150 °C are revealed in Figure S11. It can be seen that a decrease in relaxation time did not lead to a significant increase in permanent deformation, indicating that the balance between relaxation and creep performance could be achieved via the subtle design of vitrimer networks.

**Figure 11:**
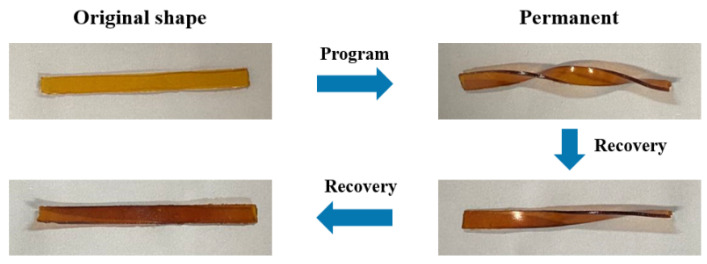
Shape-changing process of vitrimer 4 at 150 °C.

In addition, the plasticity of our designed vitrimers caused by bond exchange reactions could be confirmed via shape-changing experiments (Figure 11). The sample was deformed by the application of an external force, and the shape was maintained with heat-resistant tapes. After soaking at 150 °C for 30 min, the sample was cooled to room temperature. During the shape-changing process, dynamic disulfide linkages allowed the polymer chains to relax at high temperature, and the applied stress could be adapted by the network rearrangement, resulting in the vitrimer producing a new shape after cooling to room temperature [48].

### 3.5. Recycling Properties of PB Vitrimers

**Figure 12:**
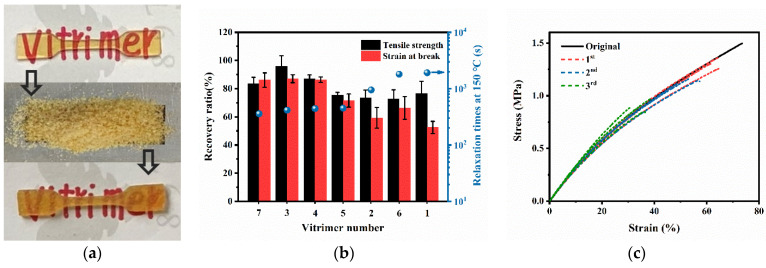
(**a**) Recycling schematic of PB vitrimers; (**b**) recovery ratios of mechanical properties for PB vitrimers after recycling; (**c**) stress–strain curves of original and recycled vitrimer 4.

Compared with traditional vulcanized rubbers, dynamically covalently crosslinked elastomers possess good processability, similar to thermoplastics, owing to the activated network rearrangement. The cured polybutadiene was ground into small particles and then hot-pressed at 150 °C for 30 min to obtain a recycled sample (Figure 12a). Next, tensile experiments were carried out to assess the recycling ability. The dynamic nature of disulfide bonds endowed the system with recyclability. Due to the thermoactivated disulfide exchange, PB vitrimers exhibited good recovery of the mechanical properties (Figure 12b and Figure S12). Nevertheless, vitrimers 1, 2, and 6 revealed relatively lower recovery ratios, compared with the other PB vitrimers. This may be ascribed to their lower relaxation rates, which restricted sufficient disulfide exchange. The stress–strain curves of vitrimer 4, a representative example, after three remolding iterations are revealed in Figure 12c. It can be seen that its mechanical properties could be maintained after several recycling times. However, they exhibited a decline with the increase in recycling steps. In fact, long-term exposure to high temperature would cause thermal degradation in polymer chains, restricting the recovery of mechanical properties. The reduction in T_d_ confirmed the occurrence of thermal degradation in the recycled sample (Figure S13a). Furthermore, irreversible covalent crosslinks might be generated under the thermal stimuli. The long-term exposure to high temperature necessary for remolding favored some side reactions, such as oxidation, resulting in insufficient network rearrangement [34]. Therefore, the mechanical properties weakened with the increasing recycling cycles. On the other hand, the FT-IR spectrum of recycled vitrimer 4 was basically unchanged, indicating a constant network structure after hot processing (Figure S13b). In other words, the influence of side reactions caused by the high temperature on the network integrity was limited.

To further reveal the importance of disulfide bonds for recycling, the remolding experiment was performed with the DA-cured sample as a control (Figure S14). Obviously, the control sample exhibited a poor reprocessing ability, indicating that disulfide units were necessary for physical recycling in our designed system.

## 4. Conclusions

In summary, PB vitrimers with different structures were prepared using epoxy groups and disulfide-containing dicarboxylic acids through esterification. In particular, the epoxidation degree of polybutadiene could be precisely controlled by the reaction-controlled phase-transfer catalysis. The network crosslinking density was tuned by varying the epoxy ratio, crosslinker length, and crosslinker molar content. In comparison, the latter two network parameters showed a more significant influence. The good stability of disulfide bonds at room temperature preserved the physical and mechanical properties of the resulting rubber. Through regulating the crosslinking density, the tensile strength and elongation at break of PB vitrimers could be manipulated. Due to the network rearrangement activated by disulfide exchange, PB vitrimers exhibited substantial relaxation behavior at high temperatures, and their mechanical properties could be maintained after recycling. The activation energy required to alter the network topology increased as the crosslinking density increased. Interestingly, it could be reasonable to deduce that, in a narrow range of crosslinking density, the relaxation properties mainly depended on the side-group effects. The values of activation energies decreased with an increase in the epoxidation degree, because the increasing epoxy groups improved the chance of an effective exchange of disulfide units. Overall, this work demonstrated that the designed rubber possessed good reprocessing ability, even at high crosslinking densities, and a subtle network design was provided, which could allow manipulating properties via simple physical and chemical controls.

## Figures and Tables

**Table 1:** The compositions of PB vitrimers.

Vitrimer	Epoxy Ratio % ^a^	Crosslinker Length	Crosslinker Molar Content % ^d^
1	10	C_8_ ^b^	1
2	15	C_8_	1
3	25	C_8_	0.5
4	25	C_8_	1
5	25	C_8_	1.5
6	25	C_6_ ^c^	1
7	35	C_8_	1

^a^ Relative to the diene group of PB; ^b^ DTBA (C_8_H_14_O_4_S_2_); ^c^ DTPA (C_6_H_10_O_4_S_2_); ^d^ relative to the monomers of EPBx.

**Table 2:** The physical properties of PB vitrimers.

Vitrimer	Parameters	T_d_ (°C)	T_g_ (°C) ^a^	T_δ_ (°C) ^b^	G’ at 30 °C (MPa)	*v_e_* (10^−4^ mol·cm^−3^)
	**Epoxy ratio**					
1	10	340	−96.7	−85.5	2.72	3.93
2	15	341	−92.7	−79.9	2.92	4.15
4	25	345	−84.1	−69.6	2.95	4.52
7	35	348	−71.7	−45.6	3.09	4.76
	**Crosslinker length**					
4	C_8_	345	−84.1	−69.6	2.95	4.52
6	C_6_	362	−82.6	−66.2	3.34	5.93
	**Crosslinker molar content**					
3	0.5	341	−86.2	−70.1	2.47	3.14
4	1.0	345	−84.1	−69.6	2.95	4.52
5	1.5	337	−82.1	−69.3	4.36	6.40

^a^ Determined by DSC; ^b^ determined by DMA.

## Data Availability

Exclude this statement.

## Supplementary Materials

The following are available online at www.mdpi.com/article/10.3390/polym13234157/s1: Preparation of epoxidized polybutadiene (EPBx); Figure S1. The epoxidation of polybutadiene via reaction-controlled phase-transfer catalysis; Figure S2. 1H-NMR spectra of polybutadiene with different epoxy ratios; Figure S3. FT-IR spectra of PB vitrimers; Figure S4. Raman spectra of vitrimer 4 and EPB25-DA; Figure S5. Crosslinking profiles of PB vitrimers with the modification of network parameters; Figure S6. Isothermal TGA profiles of PB vitrimers in air at 150 °C for 2 h; Figure S7. The relationship between mechanical properties and crosslinking density; Figure S8. Typical stress–strain curves of vitrimer 4 and EPB25-DA; Figure S9. Stress relaxation curves of vitrimers; Figure S10. Creep recovery curves of PB vitrimers; Figure S11. The relationship between permanent deformation and relaxation times at 150 °C; Figure S12. Typical stress–strain curves of PB vitrimers; Figure S13. (a) TGA of original and recycled vitrimer 4; (b) FT-IR spectra of original and recycled vitrimer 4; Figure S14. Typical stress–strain curves of original and recycled EPB25-DA; Table S1. The mechanical properties of PB vitrimers.
