# Supplementary material for: Polybutadiene Vitrimers with Tunable Epoxy Ratios: Preparation and Properties
Source: Polymers (Basel). 2021 Nov 28;13(23):4157. doi: 10.3390/polym13234157 (PMC8659766; doi:10.3390/polym13234157)
Supplement: Supplementary file 1 [file polymers-13-04157-s001.zip › polymers-1423473-supplementary.pdf]

Supplementary Materials

Polybutadiene Vitrimers with Tunable Epoxy Ratios: Preparation and Properties

Liqian Zhu, Li Xu, Suyun Jie and Bogeng Li *

State Key Laboratory of Chemical Engineering, College of Chemical and Biological Engineering, Zhejiang University, Hangzhou 310027, China; liqianzhu@zju.edu.cn (L.Z.); 11628036@zju.edu.cn (L.X.); jiesy@zju.edu.cn (S.J.)

* Correspondence: bgli@zju.edu.cn; Tel.: +86-571-8795-2623

1. Preparation of Epoxidized Polybutadiene (EPBx)

Firstly, the cis-polybutadiene rubber was added into a flask and dissolved in DCE. The catalyst (5 wt % relative to the polybutadiene weight) dissolved in DCE were mixed with aqueous H_2O_2 solution (10, 15, 25 and 35 mol. % relative to the diene content) under magnetic stirring at 60 °C to form the liquid-liquid emulsion in another flask. Then the emulsion was added dropwise into the polybutadiene solution, and the reaction was maintained under constant stirring at 60 °C. After 4 h, the mixture was cooled at 5 °C for 15 min and centrifuged to remove the catalyst. EPBx with x the epoxy ratio (i.e., the molar ratio of [H_2O_2]/[C=C]) was obtained by precipitation with methanol, followed by vacuum-drying at 30 °C overnight.

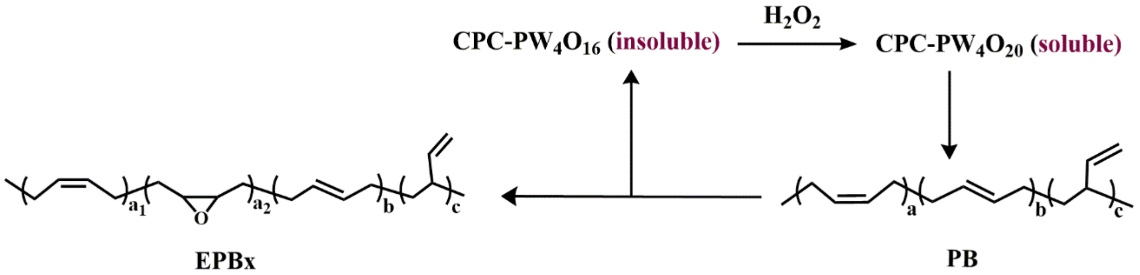

Figure S1. The epoxidation of polybutadiene via reaction-controlled phase-transfer catalysis.

2. Structure Characterization of PB Vitrimers

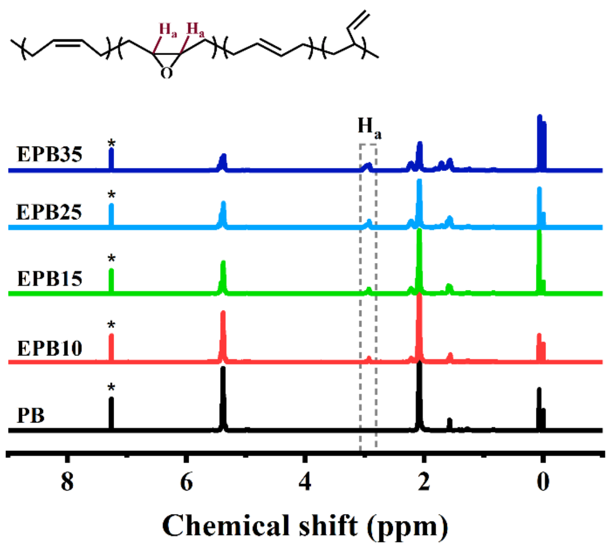

Figure S2. ^1H NMR spectra of polybutadiene with different epoxy ratios.

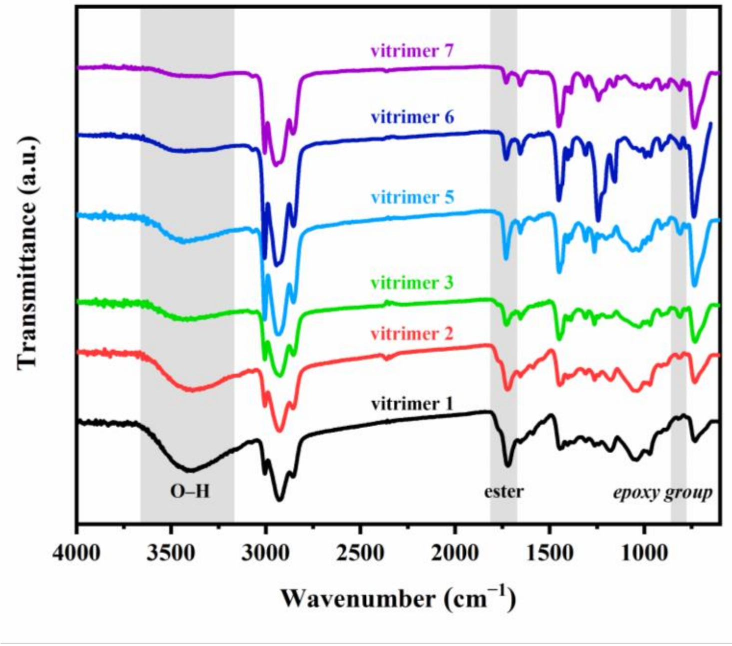

Figure S3. FT-IR spectra of PB vitrimers.

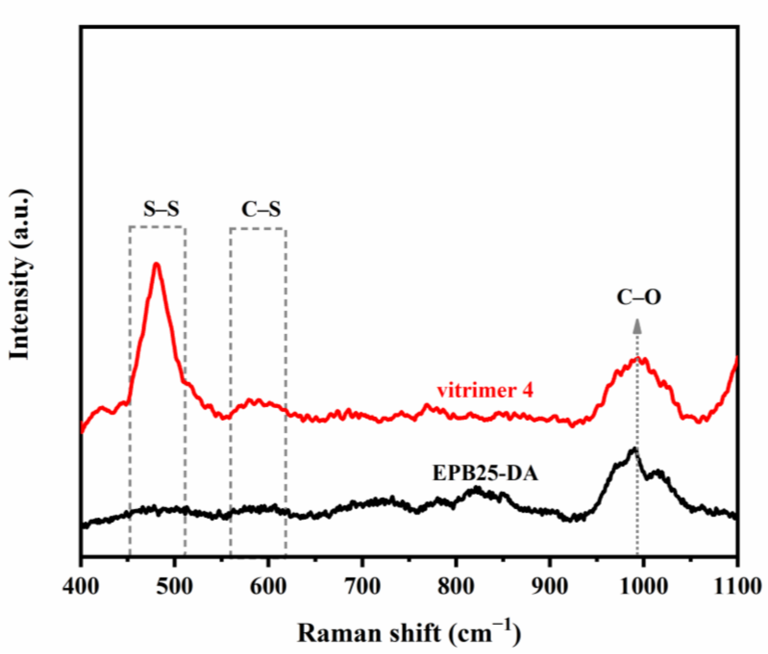

Figure S4. Raman spectra of vitrimer 4 and EPB25-DA.

3. Performance of PB Vitrimers

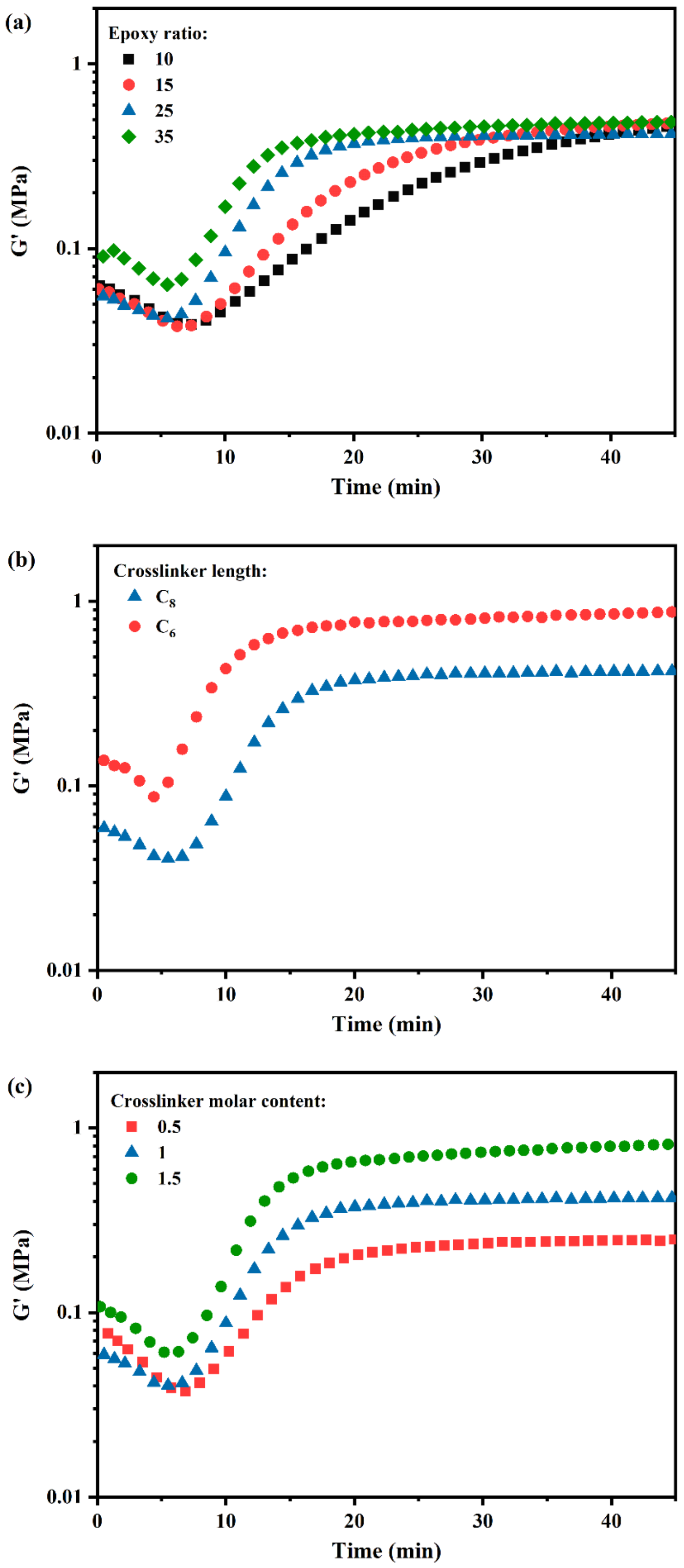

Figure S5. Crosslinking profiles of PB vitrimers with the modification of network parameters: (a) epoxy ratio, (b) crosslinker length, (c) crosslinker molar content.

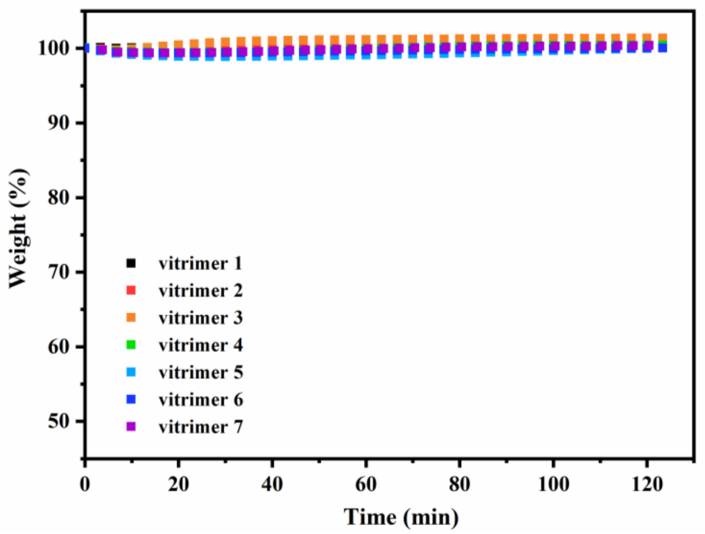

Figure S6. Isothermal TGA profiles of PB vitrimers in air at 150 °C for 2 h.

Table S1 The mechanical properties of PB vitrimers

Vitrimer	Parameters	υ_e (10^{-4} mol/cm^{-3})	Tensile strength (MPa)	Strain at break (%)
	**Epoxy ratio**			
1	10	3.93	1.26 ± 0.06	79.7 ± 7.7
2	15	4.15	1.29 ± 0.06	74.3 ± 0.4
4	25	4.52	1.42 ± 0.06	70.6 ± 2.2
7	35	4.76	1.44 ± 0.09	64.1 ± 7.5
	**Crosslinker length**			
4	C_8	4.52	1.42 ± 0.06	70.6 ± 2.2
6	C_6	5.93	1.44 ± 0.07	65.5 ± 2.2
	**Crosslinker molar content**			
3	0.5	3.14	1.33 ± 0.06	117.6 ± 15.2
4	1.0	4.52	1.42 ± 0.06	70.6 ± 2.2
5	1.5	6.40	1.49 ± 0.10	65.7 ± 7.6

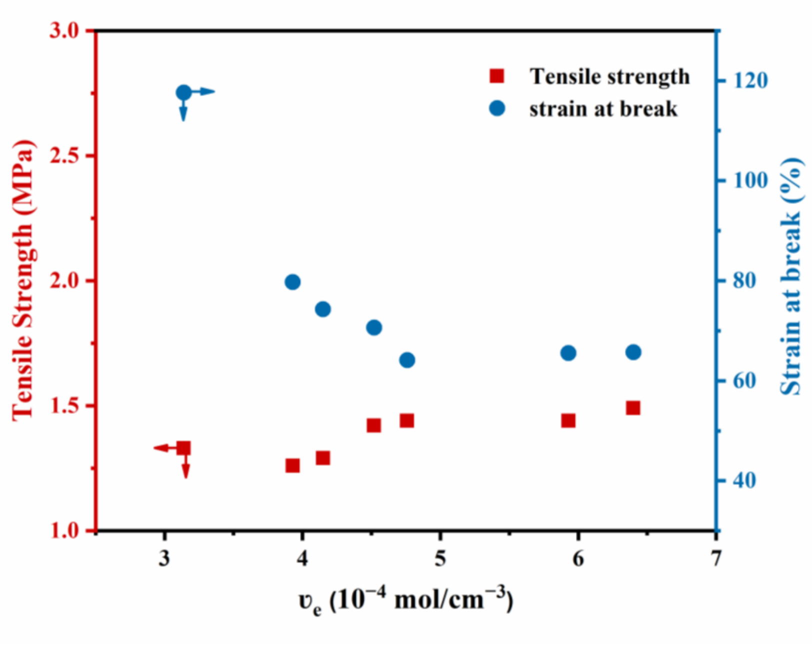

Figure S7. The relationship between mechanical properties and crosslinking density.

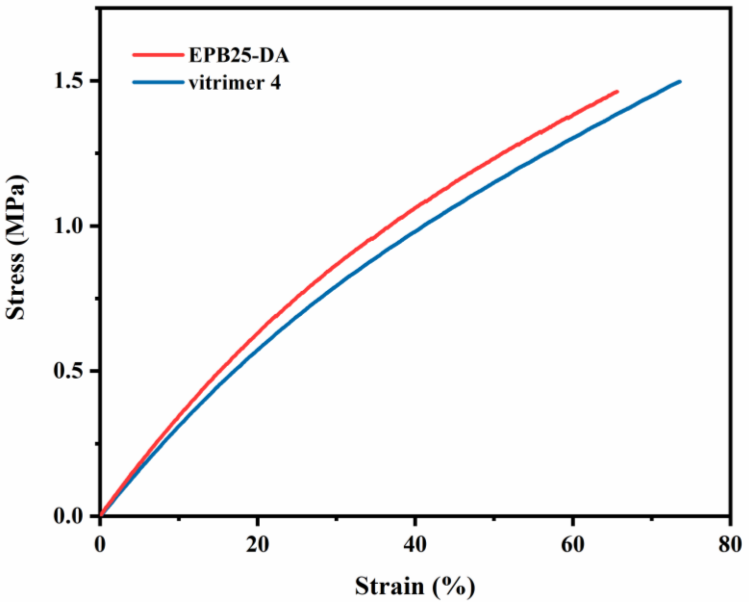

Figure S8. Typical stress−strain curves of vitrimer 4 and EPB25-DA.

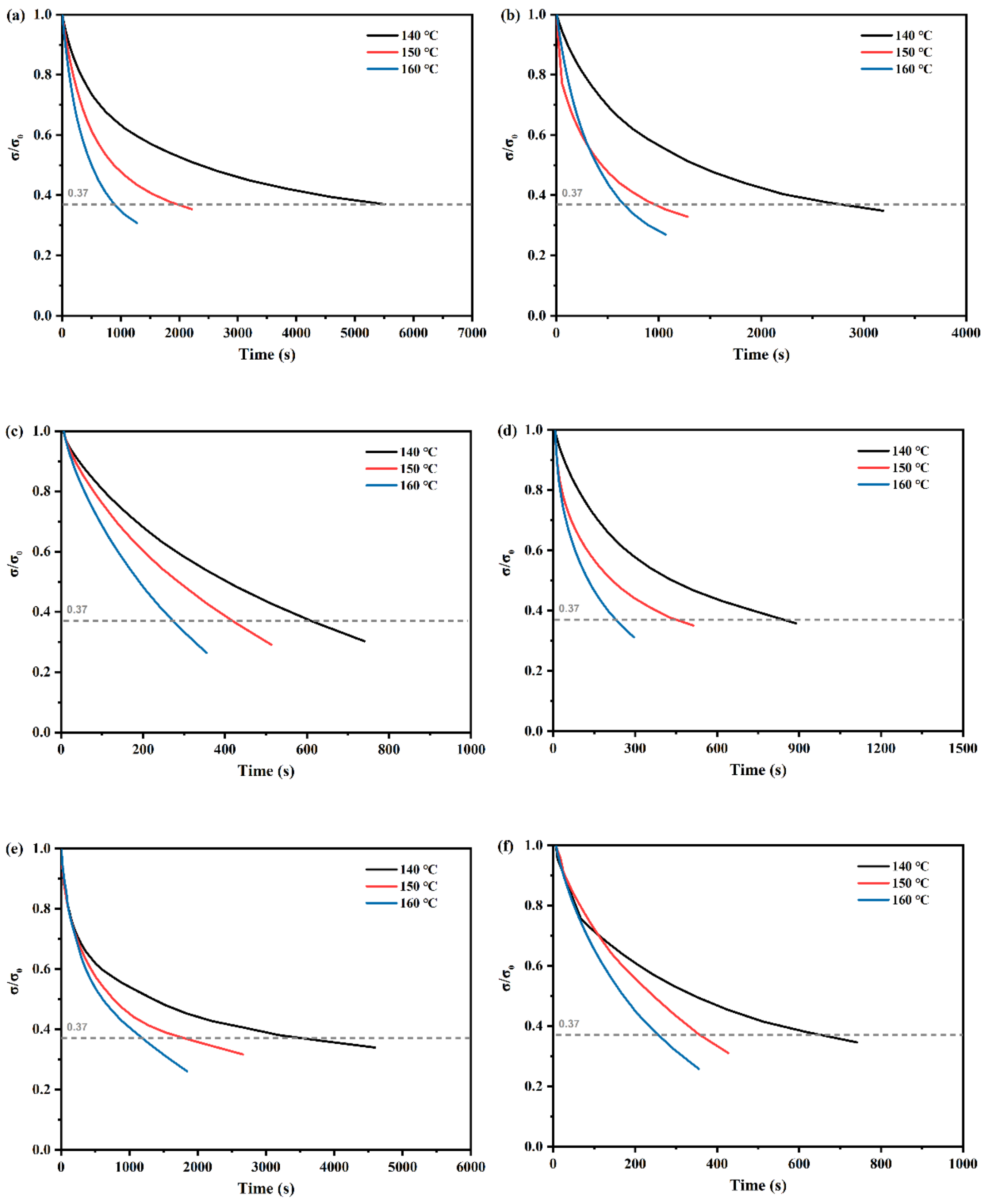

Figure S9. Stress relaxation curves of (a) vitrimer 1, (b) vitrimer 2, (c) vitrimer 3, (d) vitrimer 5, (e) vitrimer 6, (f) vitrimer 7.

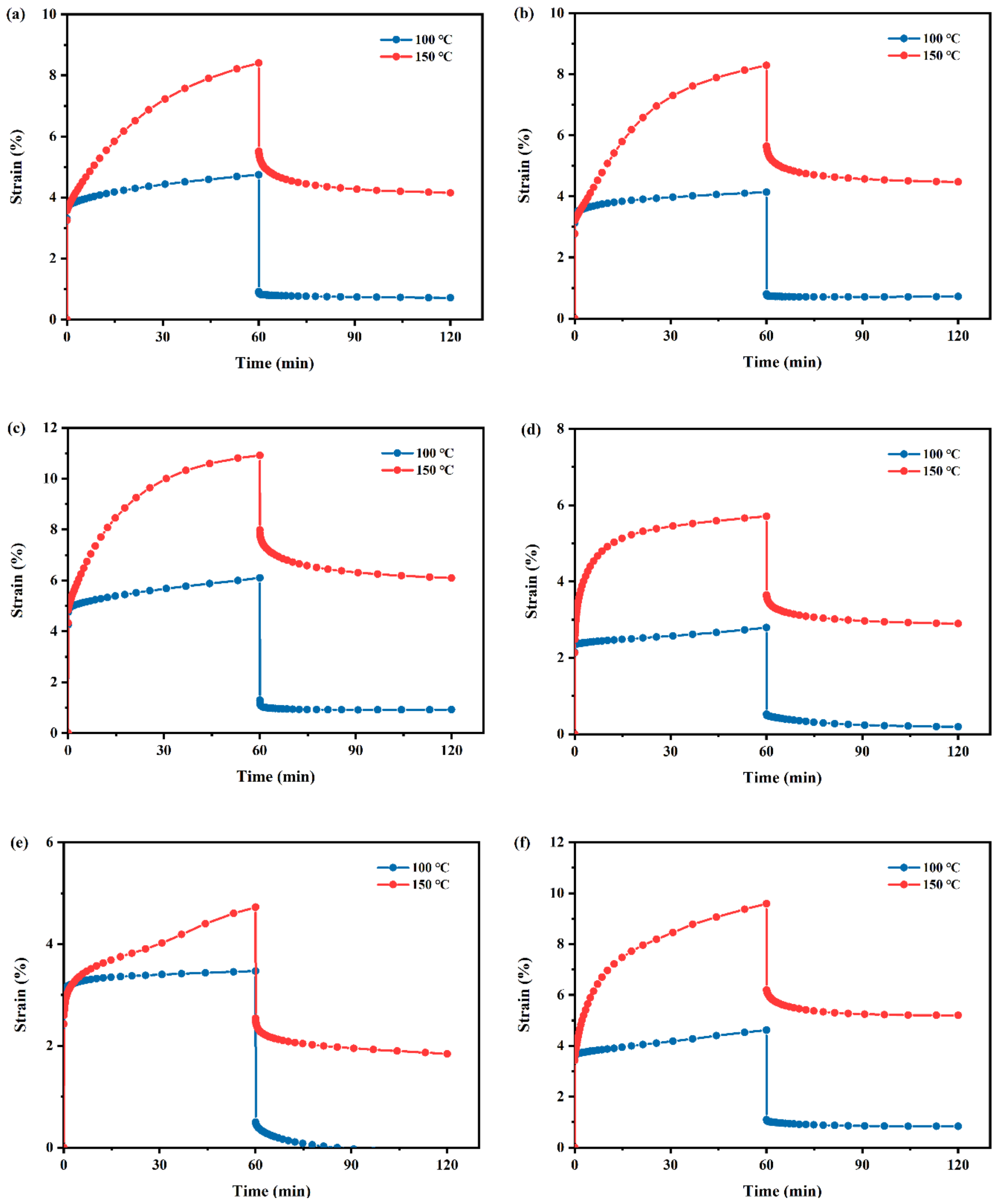

Figure S10. Creep recovery curves of PB vitrimers: (a) vitrimer 1; (b) vitrimer 2; (c) vitrimer 3; (d) vitrimer 5; (e) vitrimer 6; (f) vitrimer7.

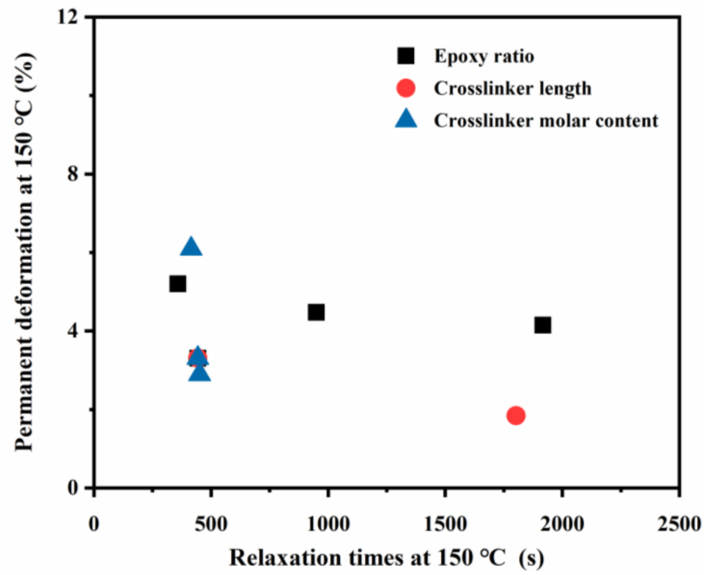

Figure S11. The relationship between permanent deformation and relaxation times at 150 °C.

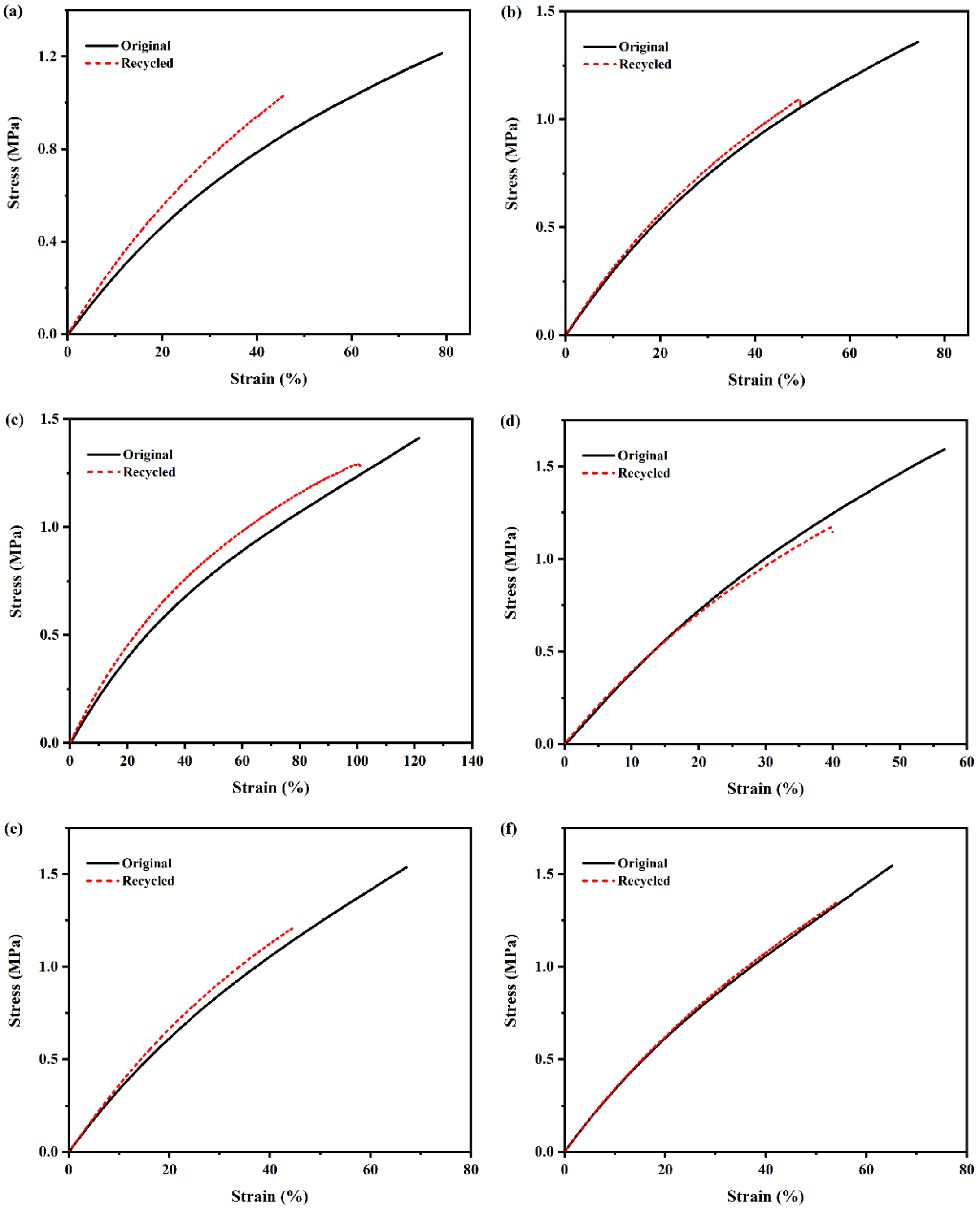

Figure S12. Typical stress−strain curves of PB vitrimers: (a) vitrimer 1; (b) vitrimer 2; (c) vitrimer 3; (d) vitrimer 5; (e) vitrimer 6; (f) vitrimer 7.

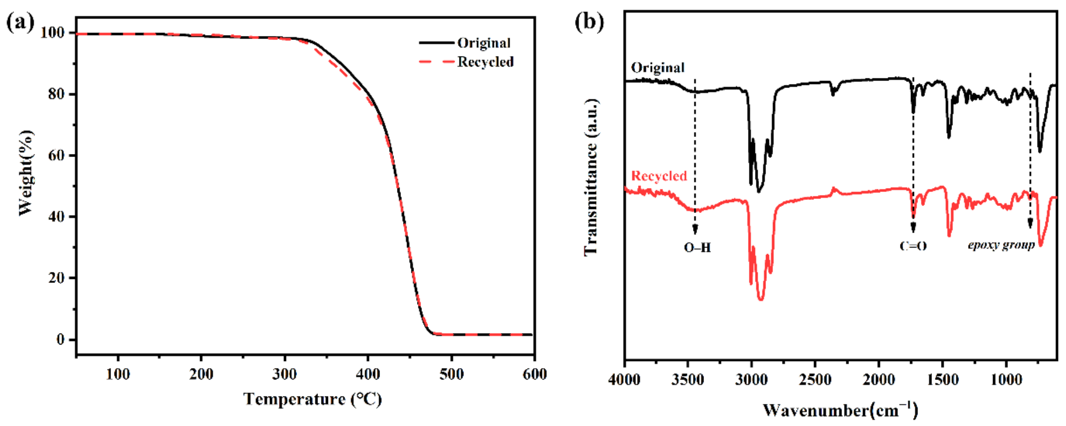

Figure S13. (a) TGA of original and recycled vitrimer 4. (b) FT-IR spectra of original and recycled vitrimer 4.

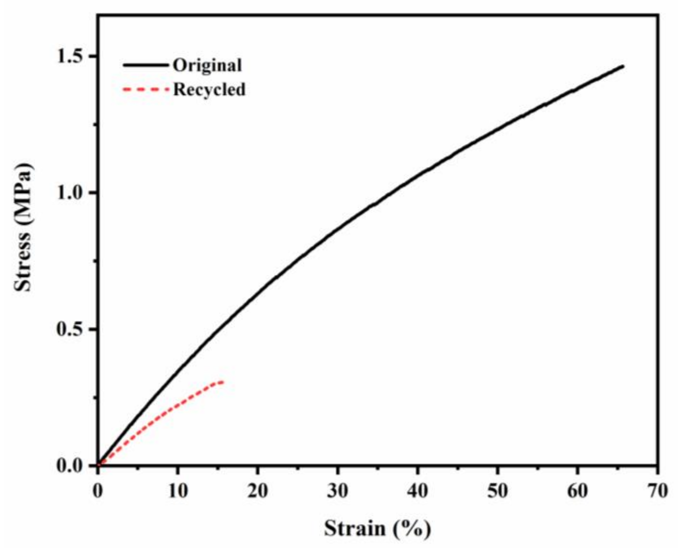

Figure S14. Typical stress–strain curves of original and recycled EPB25-DA.
